# Supplementary figures and images for: The Use of Dansyl Chloride to Probe Protein Structure and Dynamics
Source: Int J Mol Sci. 2025 Jan 8;26(2):456. doi: 10.3390/ijms26020456 (PMC11765030; doi:10.3390/ijms26020456)

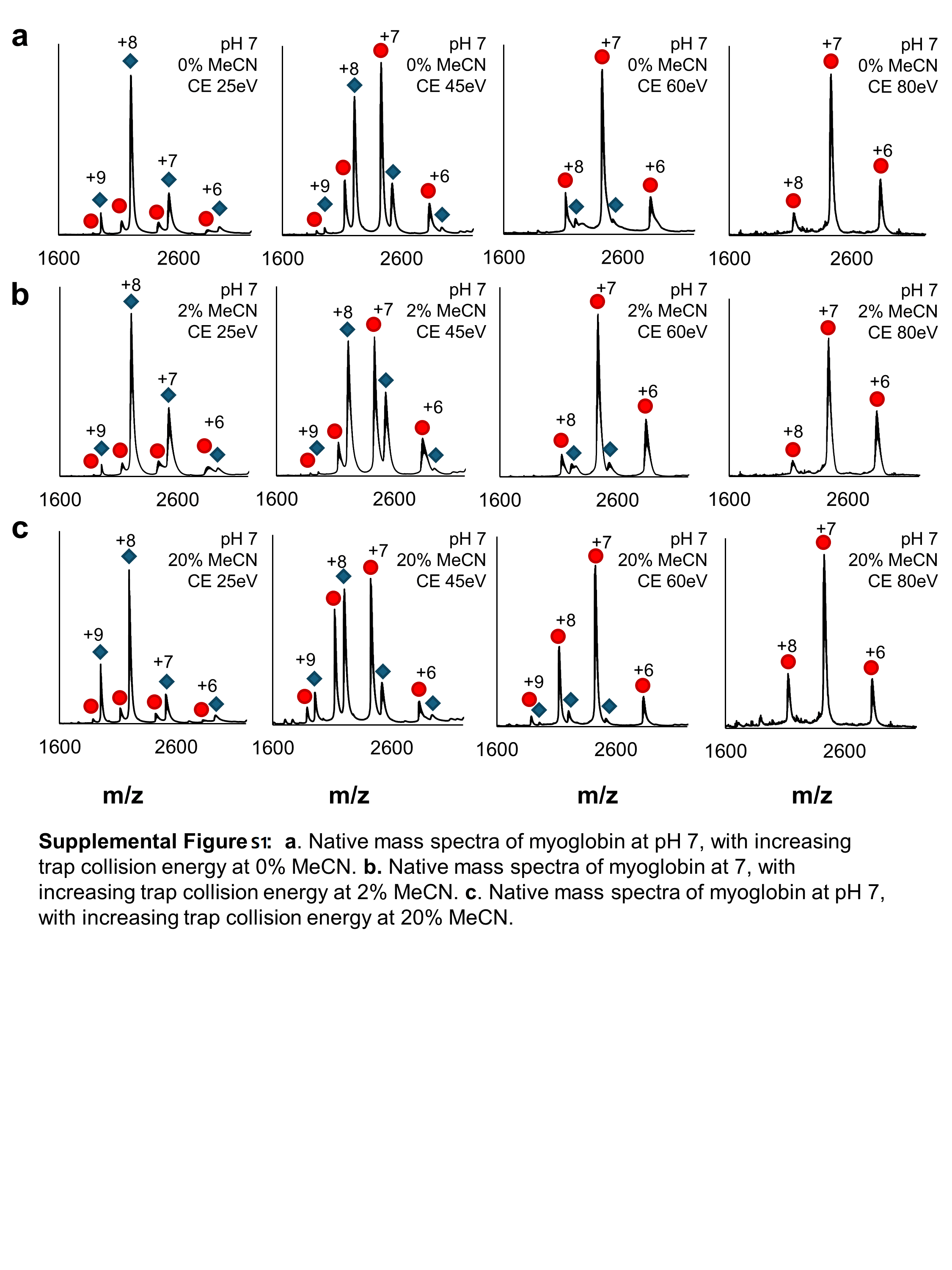

Supplement: Supplementary file 1 [file ijms-26-00456-s001.zip › Dansyl_Chloride_Supplemental_Figure_S1.png]

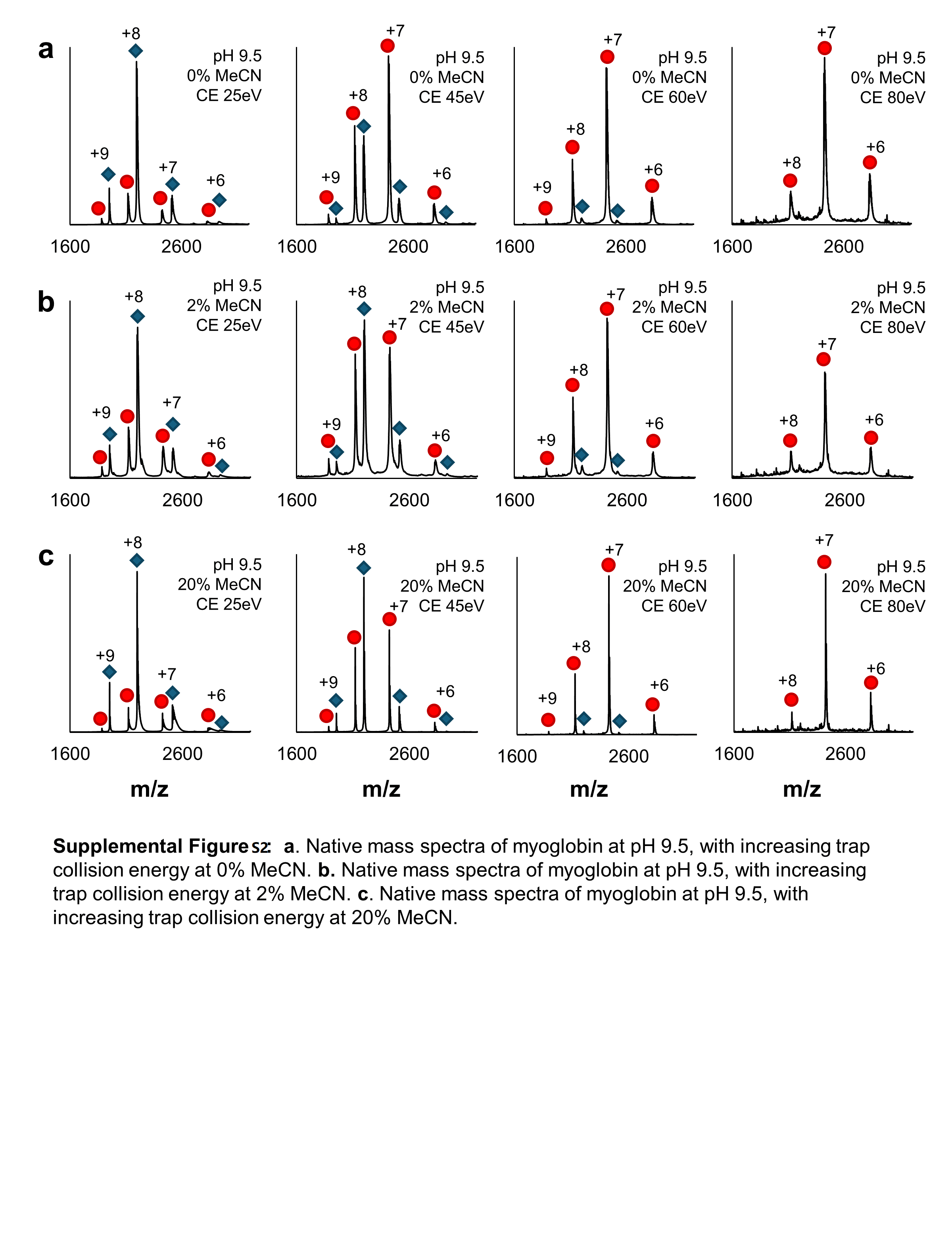

Supplement: Supplementary file 1 [file ijms-26-00456-s001.zip › Dansyl_Chloride_Supplemental_Figure_S2.png]

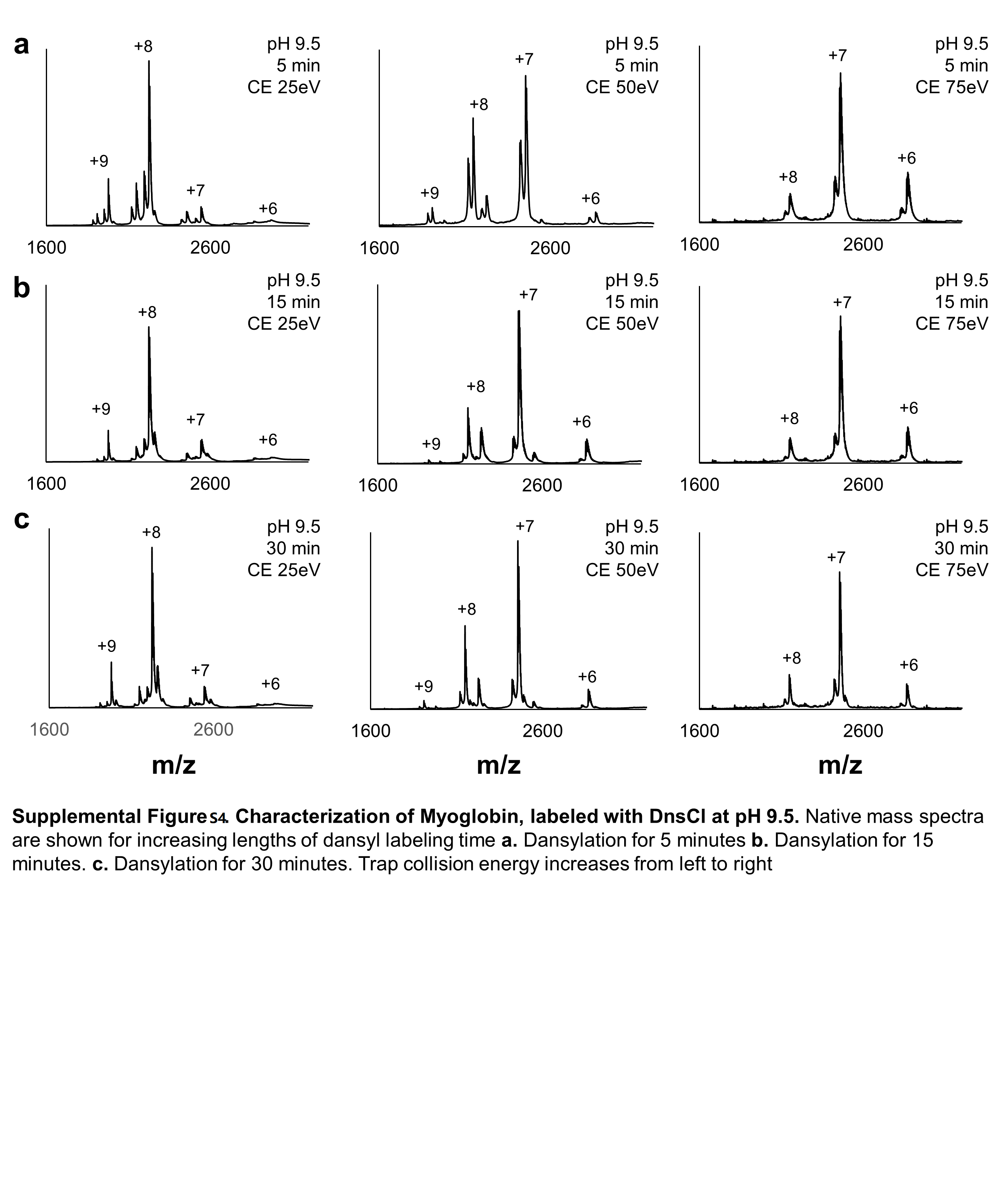

Supplement: Supplementary file 1 [file ijms-26-00456-s001.zip › Dansyl_Chloride_Supplemental_Figure_S4.png]

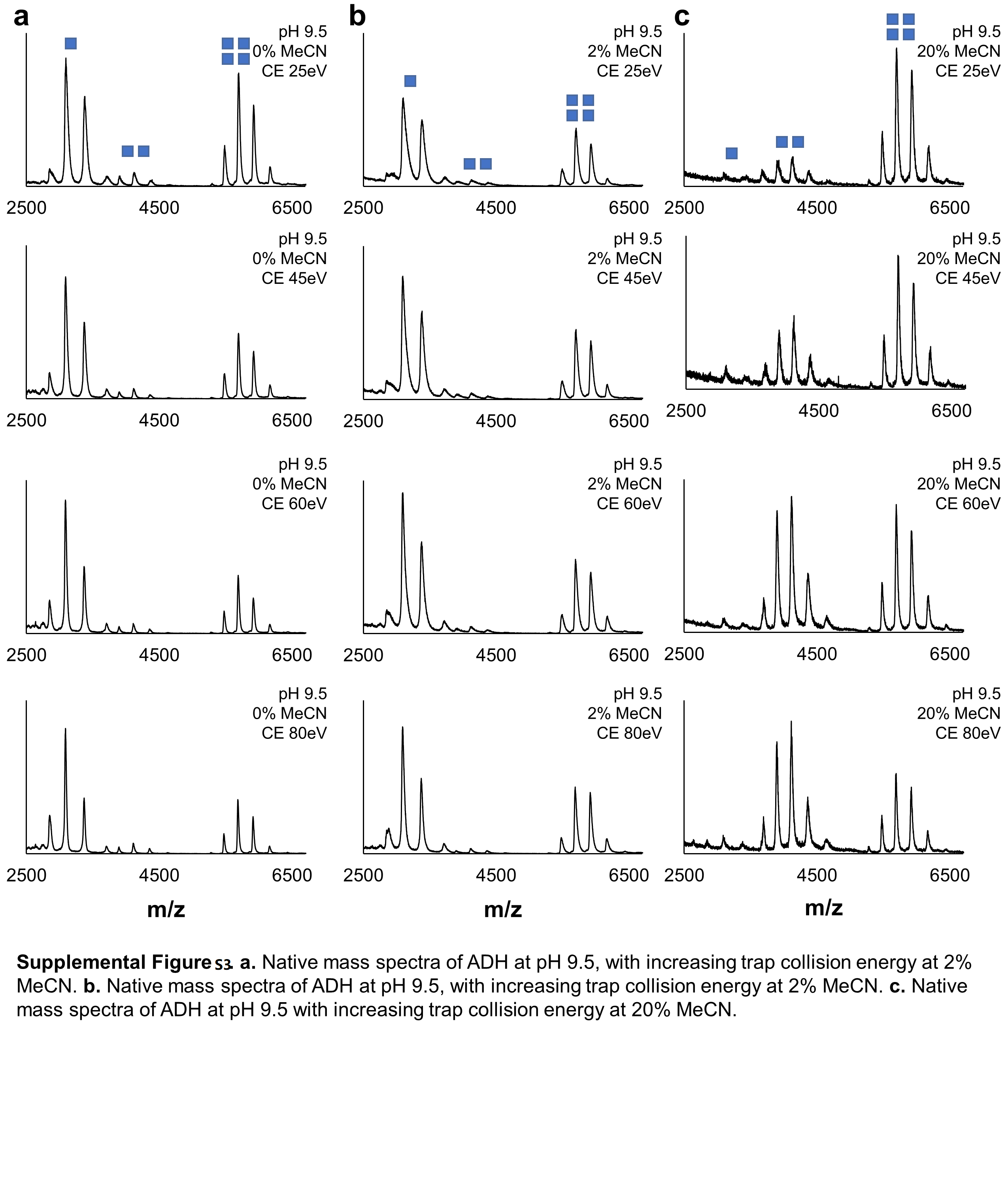

Supplement: Supplementary file 1 [file ijms-26-00456-s001.zip › Supplemental_Figure_S3.tif]
